# A Sight on Single-Cell Transcriptomics in Plants Through the Prism of Cell-Based Computational Modeling Approaches: Benefits and Challenges for Data Analysis

**DOI:** 10.3389/fgene.2021.652974

**Published:** 2021-05-21

**Authors:** Aleksandr Bobrovskikh, Alexey Doroshkov, Stefano Mazzoleni, Fabrizio Cartenì, Francesco Giannino, Ulyana Zubairova

**Affiliations:** ^1^Laboratory of Plant Growth Biomechanics, Institute of Cytology and Genetics Siberian Branch of Russian Academy of Sciences (SB RAS), Novosibirsk, Russia; ^2^Department of Agricultural Sciences, University of Naples Federico II, Naples, Italy; ^3^Department of Natural Sciences, Novosibirsk State University, Novosibirsk, Russia

**Keywords:** single-cell transcriptomics, cell-based computational models, plant morphogenesis, hybrid modeling approach, modeling software, bioimaging, spatial gene expression maps, systems biology

## Abstract

Single-cell technology is a relatively new and promising way to obtain high-resolution transcriptomic data mostly used for animals during the last decade. However, several scientific groups developed and applied the protocols for some plant tissues. Together with deeply-developed cell-resolution imaging techniques, this achievement opens up new horizons for studying the complex mechanisms of plant tissue architecture formation. While the opportunities for integrating data from transcriptomic to morphogenetic levels in a unified system still present several difficulties, plant tissues have some additional peculiarities. One of the plants’ features is that cell-to-cell communication topology through plasmodesmata forms during tissue growth and morphogenesis and results in mutual regulation of expression between neighboring cells affecting internal processes and cell domain development. Undoubtedly, we must take this fact into account when analyzing single-cell transcriptomic data. Cell-based computational modeling approaches successfully used in plant morphogenesis studies promise to be an efficient way to summarize such novel multiscale data. The inverse problem’s solutions for these models computed on the real tissue templates can shed light on the restoration of individual cells’ spatial localization in the initial plant organ—one of the most ambiguous and challenging stages in single-cell transcriptomic data analysis. This review summarizes new opportunities for advanced plant morphogenesis models, which become possible thanks to single-cell transcriptome data. Besides, we show the prospects of microscopy and cell-resolution imaging techniques to solve several spatial problems in single-cell transcriptomic data analysis and enhance the hybrid modeling framework opportunities.

## 1. Introduction

Modern biology is going through the era of big data and omics technologies. Single-cell sequencing (SCS) is one of the breakthroughs and rapidly developing technologies. This technology’s value is difficult to overestimate since it allows one to describe with high accuracy the trajectories of cell development and characterize individual cell types (Trapnell, 2015). A targeted study of isolated cells is of particular importance in the context of systems biology, as demonstrated on root hair cells (Hossain et al., 2015). The main steps of SC analysis include cellular dissociation, single-cell RNA sequencing (scRNA-seq), dimensionality reduction, clustering, and reconstruction of the developmental trajectories. McFaline-Figueroa et al. (2020) provide currently available techniques for such kind of analysis. However, such a data-driven approach provides only a partial understanding of the developmental processes for different cell types since it includes only the molecular level.

Thus, a combination of microscopy methods (Li et al., 2014) and imaging techniques (Omari et al., 2020) could provide a new level of understanding the developmental processes. In turn, the combination of high-precision SCS approaches with high-quality microscopic data can be integrated into mathematical models describing morphogenesis. Therefore, we believe that current methods for processing SC data should be coupled with morphological data on a tissue level and computational frameworks describing tissue development. Such a systemic-biological cycle will allow researchers to find out the essential spatiotemporal regulators of morphogenetic processes and provide an *in silico - in vivo* verification of emerging hypotheses.

**Figure 1:**
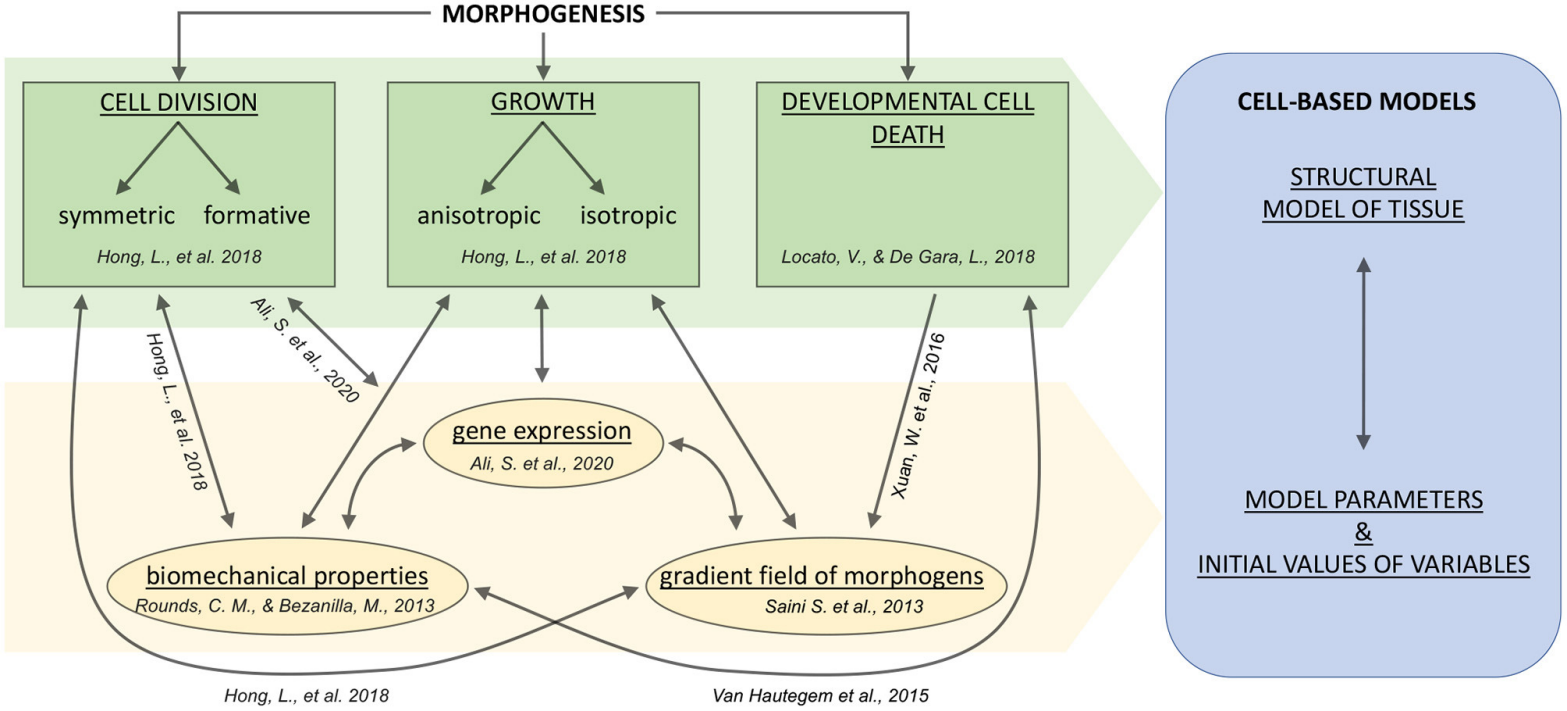
A general scheme for systems biological and modeling concepts of plant tissue morphogenesis including cell growth and division, and developmental PCD (plant cell death). Arrows indicate the relationships between fundamental cell fate and intracellular processes. The cell fate processes are indicated in green; the intracellular processes or properties are indicated in yellow. The blue box indicates the significant components of the cell-based modeling approach. References correspond to theoretical articles briefly explained in the text.

The relationship between growth characteristics of individual cells and organogenesis was noted in the work of Hong et al. (2018). In particular, it was shown that growth rate and growth direction significantly affect organ developmental processes, and, therefore, could determine the invariant organ formations. Consequently, it is essential to study cells’ individual characteristics to create a holistic picture of morphogenetic processes at the tissue and organ levels. The main drivers of morphogenesis are shown schematically below, in Figure 1. Stem cells can divide, either symmetrically or with precise daughter-cell size ratio, the so-called formative divisions, which are fundamental determinants in the processes of morphogenesis Smolarkiewicz and Dhonukshe (2013). Also, the emergence of cellular patterns forming tissues significantly depends on the anisotropic cell growth biomechanics, which occurs, in particular, in tip-growing cells (Rounds and Bezanilla, 2013).

In addition to the mechanical factors influencing growth, it is known that the formation of apical meristems (which are the niches of undifferentiated stem cells) is complex and includes molecular, hormonal and epigenetic levels of regulation (Ali et al., 2020). Moreover, the realization of the cell death program is known to be a stimulating factor for hormone signaling in developmental processes (Xuan et al., 2016), and a detailed overview and classification of plant cell death can be found in Locato and De Gara (2018).

The multilevel nature of morphogenetic processes increases the need for systemic biological research that integrates multilevel data. For example, a combination of advanced microscopy, sequencing, and artificial intelligence allows us to elaborate on the initial plant cell atlas (Rhee et al., 2019). We also see great potential in complex studies and cell-based models describing morphogenetic processes.

This review aims to show how the combination of SC data, morphometric data, and cell-based models will expand our understanding of tissue and organ morphogenesis. We discuss the possibilities and prospects of such an integrative approach for solving reverse problems, including SC data and tissue imaging coupled with cell-based morphogenesis models. Finally, we consider available tools for cell-based models and present our cell-based modeling framework for morphogenetic processes. This algorithm is iterative and includes six main steps: (i) model formulation; (ii) design experiments to obtain microscopy and scRNA-seq data; (iii) obtaining experimental data; (iv) data analysis; (v) data integration into a hybrid (discrete-continuous) mathematical model of morphogenesis; (vi) model validation and verification.

## 2. Existing Approaches to the Analysis of Single-Cell Data and Their Potential for Cell-Based Models

Characterizing the plant cell fate and ontogenesis using SC technologies is a novel and promising approach for getting high-resolution genomic data that reveals new facts about various cell types. The first SC transcriptomic experiments have been carried out for the model plant *A. thaliana* in 2019. For *A. thaliana*, most of SC studies were conducted on root cells (Denyer et al., 2019; Jean-Baptiste et al., 2019; Ryu et al., 2019; Shulse et al., 2019; Turco et al., 2019; Zhang et al., 2019; Farmer et al., 2021). Whereas, there are only two studies conducted on leaf tissues (Kim et al., 2021; Lopez-Anido et al., 2021). Thus, for all the main cell types of roots and leaves, the developmental trajectories were revealed. Also, *Zea mays*, being a representative of C4-photosynthetic cereals, is a promising object for SC experiments due to the large size its cells, which allows to easily isolate specific cells, for example, from the shoot apical meristem. To date, there are studies based on the single-cell analysis for corn tissues carried out on a shoot apex (Satterlee et al., 2020), phloem (Bezrutczyk et al., 2021), and ears (Xu et al., 2021). The first and so far only scRNA-seq on rice roots (Liu et al., 2021) revealed significant differences in the characteristics of individual cell types in comparison to the cell types of *A. thaliana*, which indicates the presence of significant species-specific differences at the cellular level. A brief summary of the currently existing Sc-experiments is given in Table 1.

**Table 1:** Summary of scRNA-seq datasets obtained for plants.

**Publication date**	**References**	**Drop-Seq platform**	**Illumina platform**	**Organism**	**Plant organ**	**Average reads per cell**	**Total genes detected**	**Expressed genes per cell**
March 2019	Denyer et al., 2019	NanoDrop	NextSeq	*A. thaliana*	Root	87.000	17.000	4.276
April 2019	Ryu et al., 2019	10X Genomics	HiSeq 4000	*A. thaliana*	Root	75.000	22.000	5.000
May 2019	Zhang et al., 2019	10X Genomics	NovaSeq	*A. thaliana*	Root	40.000	23.161	1.875
May 2019	Shulse et al., 2019	Drop-seq v. 3.1	HiSeq 2500, HiSeq 4000, NextSeq	*A. thaliana*	Root	>1,000 UMI	20.464	1.549
May 2019	Jean-Baptiste et al., 2019	10X Genomics	NextSeq 500	*A. thaliana*	Root	19.000	22.000	2.445
July 2019	Turco et al., 2019	Drop-seq v. 3.1	NextSeq	*A. thaliana*	Root	NA	21.603	NA
April 2021	Lopez-Anido et al., 2021	10X Genomics	NextSeq500, HiSeq4000	*A. thaliana*	Leaf	70.000	NA	1.870
December 2020	Satterlee et al., 2020	Droplet microfluidics	NextSeq 500	*Zea mays*	Shoot	NA	NA	2000
January 2021	Kim et al., 2021	10X Genomics	HiSeq 2500	*A. thaliana*	Leaf	96.000	27.000	3.300
January 2021	Farmer et al., 2021	10X Genomics	HiSeq	*A. thaliana*	Root	NA	25.000	4.700
January 2021	Bezrutczyk et al., 2021	10X Genomics	HiSeq	*Zea mays*	Phloem	5,000	NA	NA
February 2021	Xu et al., 2021	10x Genomics	NextSeq 500	*Zea mays*	Ears	32.000	28.900	1800
March 2021	Liu et al., 2021	10x Genomics	HiSeq 2000	*Oryza sativa*	Roots	NA	NA	2600

There are several fundamental questions about the limitations and capabilities of the SC method (Rich-Griffin et al., 2020): How realistic is it to recreate a cell atlas using such data? Can we apply the technology to cells of any type? How to identify the main gene regulators and gene networks of development?

The problem of combining SC data from different plant species is of particular interest since the successful application of this approach can be used to create a unified developmental atlas. However, it is necessary to consider the species-specific features of tissue development and organization, which imposes certain restrictions on the joint interpretation of the exact SC data.

There is an acute lack of SC data of leaf and shoot stem cells except for *A. thaliana*. The small amount of existing SC transcriptome data is partly due to the complexity and length of the required experimentation and data analysis. In a recent overview of SC methods for plants (Lähnemann et al., 2020; Shaw et al., 2020), the authors highlight the major challenges and drawbacks of single-cell approaches: (i) gene expressing bias caused by the protoplasting procedure, (ii) unequal efficiency for extraction of different types of cells, (iii) difficulties for the reverse reconstruction of the cell atlas based on transcriptomic data, (iv) lack of data. We also want to point out that there are fuzzy boundaries between cell populations due to their connectivity and the presence of transport processes between them. Therefore, there are still several limitations to the biological interpretation of the SC data.

Thus, the classification of cell types and reverse spatial reconstruction are critical stages of SC transcriptome data analysis. This task is rather complex and requires using the original dimension of the expression data. SC data generally represent a filtered and normalized array with dimension *M* × *N*, where *M* is the number of cells with a sufficient number of reads, *N* is the number of genes with a non-zero expression. The first component that can facilitate this problem is certain developmental trajectories caused by intracellular factors that limit the space of developmental possibilities and cause their partial determinism. Such factors have a different nature: the concentration of substances and energy substrates in the cell, the concentration of hormones and morphogens, the mechanical characteristics of cells (e.g., turgor pressure, tension, and thickness of the cell wall). Unfortunately, it is currently impossible to estimate the effect of these factors and their contribution to genes’ expression. However, their presence makes it possible to identify the main differentiation genes. In general, this fact allows to carry out the procedure for reducing the dimensions of data. Depending on the data set’s complexity, it is proposed to select from 1,000 to 5,000 highly variable genes for clustering and cell classification (Luecken and Theis, 2019).

A variety of available methods and tips for single-cell data dimensionality reduction and clustering are presented in the work of Nguyen and Holmes (2019). In most cases, researchers choose t-SNE and UMAP algorithms. The large computational complexity of the t-SNE method on big datasets was eliminated by adding fast Fourier transforms (Flt-SNE, Linderman et al., 2019). Comparison of t-SNE and UMAP methods revealed that UMAP outperforms even an optimized t-SNE in the computation time; also, clustering by UMAP is the most meaningful for distinguishing between cell types (Becht et al., 2019). Before the widespread use of t-SNE and UMAP, there was a probabilistic modeling method using Bayesian mixture of factor analyzers (MFA) (Campbell and Yau, 2017), based on the assumption that changes in gene expression are a linear function of time, which allows performing the Gibbs sampling procedure. This method’s stability is inversely proportional to the number of genes with non-linear transient behavior, and its threshold was estimated in 40% of the total sample; if this threshold is exceeded, the authors recommend using the Diffusion Pseudotime (DPT) method (Haghverdi et al., 2016).

Also, machine learning demonstrates its consistency and efficiency in the analysis of SC transcriptomic data. For example, single-cell interpretation via multi-kernel learning algorithm (SIMLR) can perform dimension reduction, clustering, and visualization; this algorithm is characterized by enhanced performance and better visualization and interpretability compared to t-SNE, PCA, and zero-inflated factor analysis (ZIFA) methods (Wang et al., 2017). There are additional packages and algorithms for analyzing single-cell data, from preprocessing to data visualization; for example, on the Bioconductor platform (Amezquita et al., 2020), or the Python-based scalable toolkit SCANPY (Wolf et al., 2018).

Modeling the dynamics of gene networks is a promising approach for extracting biological facts from single-cell transcriptomics. When reconstructing such networks, it is possible to identify both transcriptional regulators and their targets. For example, a high-performance TENET protocol is based on the calculation of transfer entropy and can predict large-scale gene regulatory cascades and relationships in single-cell data (Kim et al., 2020). Also, there is SCENIC, a fast calculation Python algorithm that reconstructs the regulons (Van de Sande et al., 2020). Comparing the accuracy of calculations of gene networks by different algorithms showed that successful methods on artificial data sets are characterized by low accuracy on real data (Pratapa et al., 2020). The authors have selected three promising methods with high computational accuracy on real data: partial information decomposition and context (PIDC) (Chan et al., 2017), gene network inference with the ensemble of trees (GENIE3) (Irrthum et al., 2010), and GRNBoost2 (Moerman et al., 2019).

Elaboration of specific algorithms for using SC transcriptomic data to reconstruct developmental gene networks and identify new regulators remains a challenging issue. Databases and genetic interactions can serve as an additional source for expanding genetic networks and their verification. For example, STRING database (Szklarczyk et al., 2019) includes information about protein-protein interactions and allows to perform network reconstruction, visualization and functional enrichment analysis. Cytoscape is a suitable environment for further network visualization and addition of meta-information (Shannon et al., 2003). The functionality of this application has been significantly expanded due to the many available plugins. For example, the GeneMANIA plugin (Warde-Farley et al., 2010) allows to predict additional network elements and new connections, whereas the plugin *yFiles* (Wiese et al., 2004) provides additional tools for network layout.

Another ambitious challenge is the integration of multi-omics SC data. Ma et al. (2020) examines the capabilities of 10 SC integration tools and tests the functionality of the four most relevant ones (Giotto, MOFA, LIGER, Seurat3). It should be noted that the existing problems in the analysis and interpretation of data give rise to the rapid development of various methods and approaches to their processing. The available collection of various methods and tools for analyzing SC data is presented in this online repository. Also, pipelines and statistical methods useful for analyzing SC data are presented in the work by Petegrosso et al. (2020).

Although obtaining high-quality SC transcriptomic data for plants is a routine, standardized procedure, cell extraction processes, meaningful interpretation and verification of data are essential and non-trivial stages for the development of this technology. An important step in data validation and interpretation is the construction of mathematical cell-based models, which combines the data about concentration of morphogens and expression of genetic regulators inside the cells and “rules,” which determine intercellular communications, cellular mechanics, transport processes as well as the transition between cellular states. However, with current technology, we cannot directly use the entire array of transcriptome data to create mathematical models of morphogenesis due to the large number of dimensions. Therefore, it is important when comparing different cell types to identify the main genetic and metabolic differences and take them into account in models.

**Figure 2:**
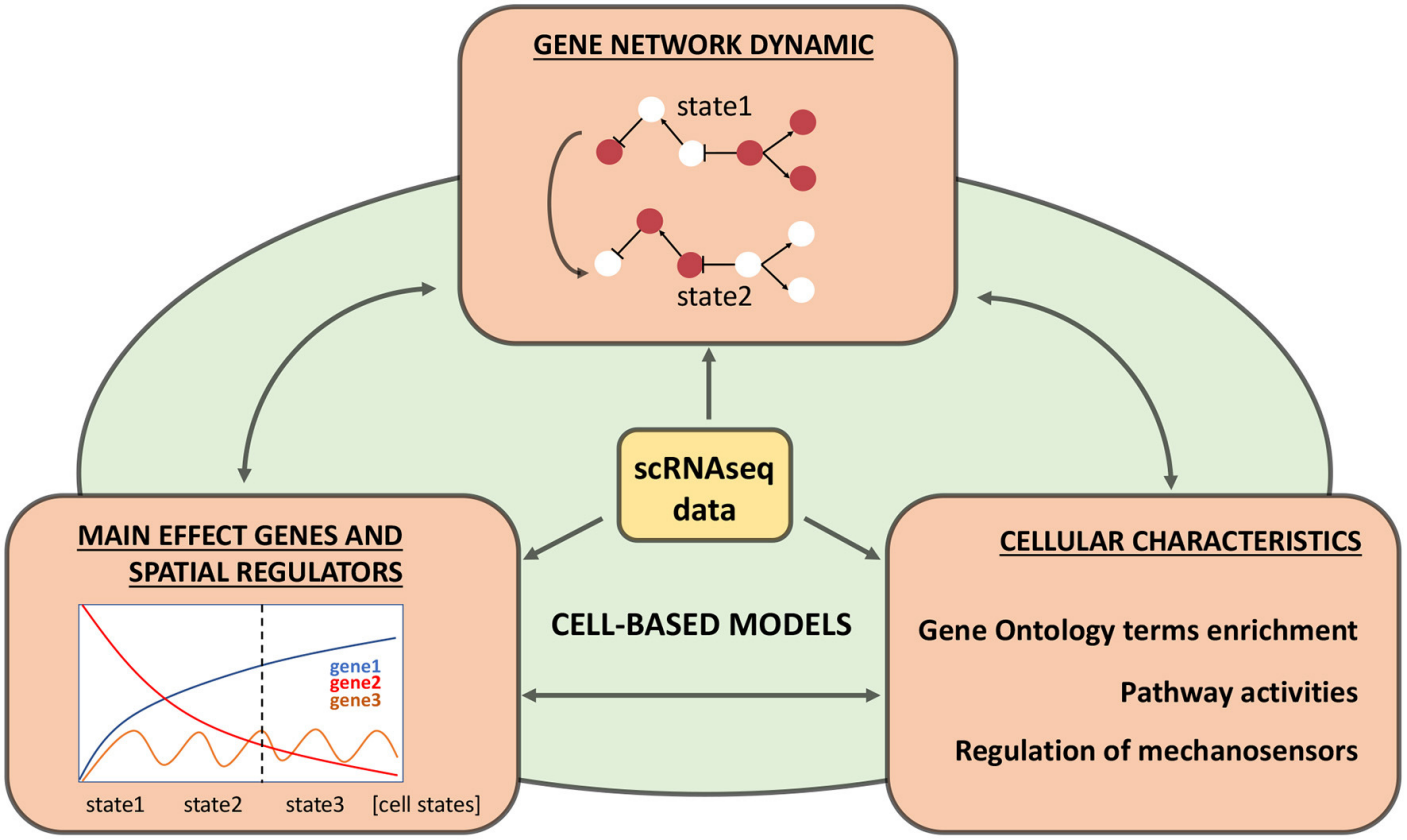
Relevant information from single-cell transcriptomics experiments for cell-based models. Three types of information are highlighted in orange blocks, their integration into the cell-based model is shown in green, and double-headed arrows indicate each block’s comparison. The central yellow block indicates original processed single-cell RNA sequencing (scRNA-seq) data.

There are a few methods, which can potentially allow researchers to use scRNA-seq data for building the cell-based models (see Figure 2):

1. Identifying crucial genes (main effect genes) and regulators which explain a lot of variance/differences between cell types.
2. Searching for novel regulatory genes, which have a spatial distribution of expression between cells of different types.
3. Reconstructing Boolean gene networks using transcriptomic data.
4. Estimation of differences in integral characteristics (such as biomass, wall thickness, concentration of metabolites).

For example, SC transcriptome data could provide some indirect estimations of the cell wall’s mechanical properties. The main mechanosensing genes are described in Du and Jiao (2020): receptor-like kinase FERONIA (FER), Leucine-rich repeat extensins (LRXs), DEFECTIVE KERNEL 1 (DEK1), and their targets of cell wall integrity pathways. Therefore, assessing these genes’ expression levels in different cell types can potentially describe their mechanosensitivity and cell wall stiffness. Thus, SC data allows the definition of cell types’ molecular characteristics, identifies regulatory subnetworks, and assesses their dynamics. These data can potentially be taken into account as parameters in cell-oriented models.

## 3. Modern Imaging Technologies for Obtaining Data on Plant Tissues With a Single-Cell Resolution

Spatial organization plays a significant role in each cell’s fate, affects transport, the direction of division, apoptosis, and the cells’ structural peculiarities. Therefore, this information is the basis for a systemic integrative study of the processes of morphogenesis.

The cells of vascular plants form a shared symplast through the cell walls, which determine the fixed position of the cells in the tissue (Vaahtera et al., 2019). In plants, cell migration is almost absent, but in some cases, cells can shift their positions relative to each other: part of the plant cell remains in its original place, while other parts of the cell grow to the new locations, moving significantly relative to other cells (Lev-Yadun, 2015).

**Figure 3:**
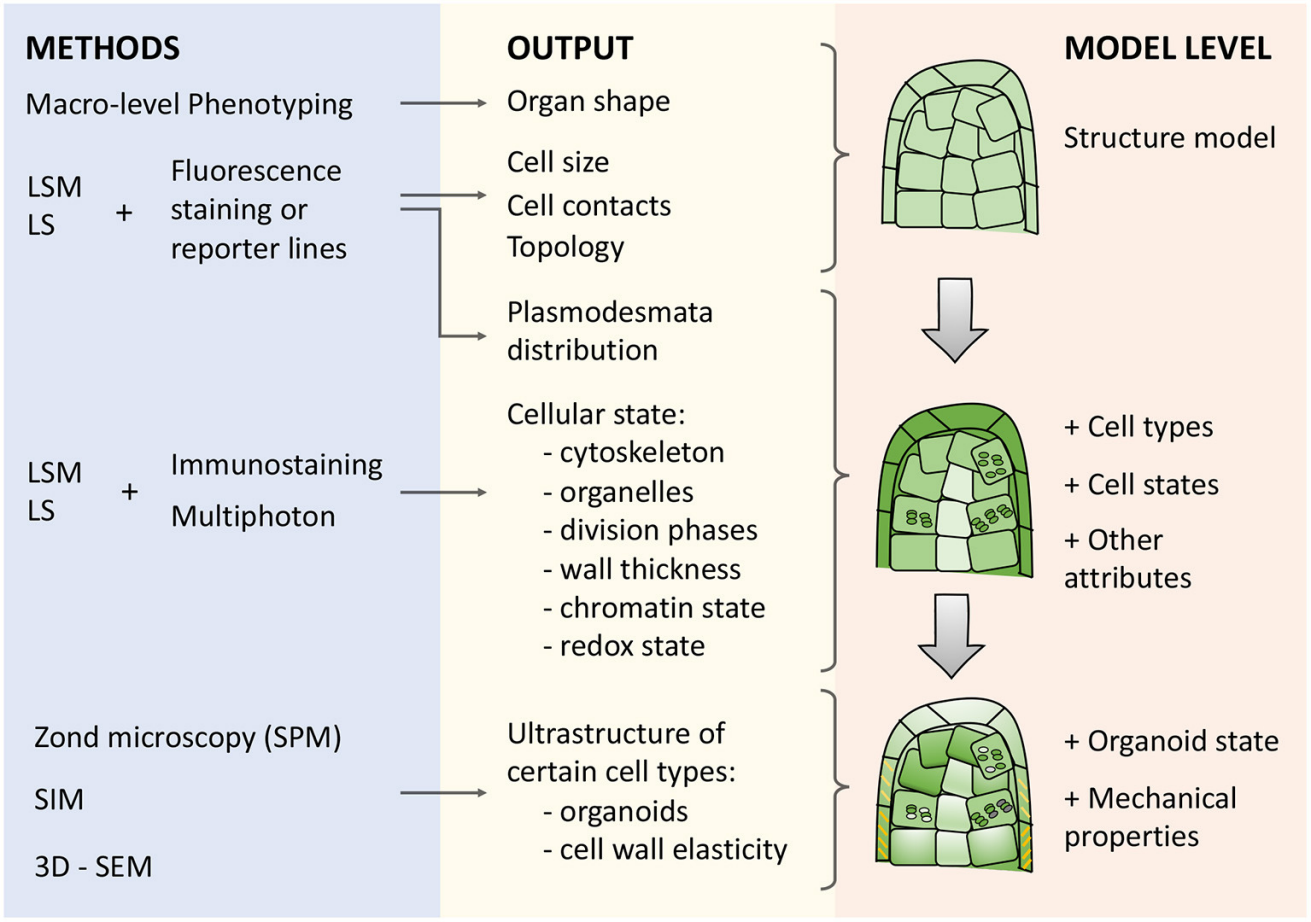
Types of microscopy techniques, their outputs, and meanings for describing morphogenetic processes in cell-based models. There are three blocks in the scheme: (i) methods (blue box), (ii) corresponding outputs (yellow box), and (iii) model levels (orange box) from structural to organoid resolution. Abbreviations used: LSM (Laser Scanning Microscopy), LS (Light-Sheet microscopy), SPM (Scanning probe microscopy), SIM (Structured Illumination Microscopy), 3D-SEM(3-Dimensional Scanning Electron Microscopy).

There are various specialized approaches for phenotyping (Figure 3): visible light, spectroscopy, infrared, fluorescence, 3D, and tomographic methods for getting plant images (Li et al., 2014). The imaging techniques for plant quantification are broadly used due to their inexpensive cost, simplicity of operation, and maintenance (Omari et al., 2020).

Reconstruction of plant architecture in terms of shape, size, and topology of cell connections (Figure 3) is an essential component to reach an integrative systemic understanding of aspects of the functioning of both individual cells and tissue as a whole (Fricker, 2016; Zubairova et al., 2019; Kerstens et al., 2020). A variety of optical tissue imaging techniques (Figure 3) currently allow access to such cellular characteristics (optical and fluorescent microscopy, laser scanning approaches, and structured lighting microscopy). Since higher plants’ organs are multilayered and volumetric, imaging techniques based on 3D analysis of a fluorescent signal, such as laser scanning microscopy, are currently among the most widespread visualization methods of cellular architecture. It allows to reconstruct the architecture of tissue and organ fragments consisting of thousands of cells (Zubairova et al., 2019) and to analyze *in vivo* large time-series for reconstructing the dynamics of development (Goh, 2019; Seerangan et al., 2020).

Together with modern image analysis methods, they provide a reliable decomposition of cell layers and assessment of cell morphological parameters (Legland et al., 2016; Erguvan et al., 2019; Zubairova et al., 2019). The number of cells reconstructed by ImageJ-plugins LSM-W^2^ (Zubairova et al., 2019), SurfCut (Erguvan et al., 2019), as well as MorphoGraphX instruments (Kerstens et al., 2020) is limited by the computer performance and technical capabilities of the microscope. They allow working on a local computer with arrays from thousands of cells, which is of a comparable order to scRNA-seq methods. The most comprehensive range of methods makes it possible to segment cells, measure cell shape parameters, and reveal the topology of cells’ connection with each other (Jackson et al., 2017).

Over the past few years, the possibility to study many entire organs through complete reconstruction at the cellular level became a significant breakthrough (Wolny et al., 2020). The root tip of *A. thaliana* is the most abundant target for scRNA-seq in plants. At the same time there are many reconstructions and 3D atlases for it (Dolan et al., 1993; Bowman, 2012; Mai et al., 2014) and even specialized software that allows displaying the various cellular characteristics into cellular ensembles, for example, the iRoCS Toolbox (Schmidt et al., 2014). *In vivo* laser scanning microscopy techniques coupled with mathematical modeling allowed describing the processes of morphogenesis for the arabidopsis root apical meristems (Mironova et al., 2012). The dynamics of the development of *A. thaliana* lateral roots are also available for visualization at the cellular level from the earliest stages of their establishment (Goh, 2019). Using confocal and multiphoton microscopy approaches, apexes and leaf primordia can also be completely reconstructed (Kiss et al., 2017; Wolny et al., 2020), as well as adult leaves (Wuyts et al., 2010) and sepals (Tauriello et al., 2015).

3D reconstruction of *A. thaliana* ovule coupled with transcriptome sequencing provides incredibly detailed data about developmental processes of this organ (Vijayan et al., 2021), which can serve as a set of reference points for further integration of future single-cell data on this organ. Simultaneously, the methods of visualization and analysis of images also allow working with plants with larger organs, for example, with *Nicotiana tabacum* roots (Pasternak et al., 2017).

Light-sheet imaging techniques allow to increase the scan depth and improve the quality of the reconstruction. These technologies, coupled with mathematical modeling, gave insights into the geometrical organization of divisions during the formation of the lateral root of *A. thaliana* (von Wangenheim et al., 2016). In particular, the first division of the cell-founders is always asymmetric and determines the formation of a layered structure, while the pattern of further cell division forms thanks to a regular change in the orientation of the division plane. Also, the technique of optical cleaning of plant tissues allows for getting deep 3D imaging and is compatible with fluorescence-based microscopy (Warner et al., 2014). The measurements of morphological characteristics of cells and their mutual arrangement allowed researchers to form a structural model of the studied organ and identify cell types (Kerstens et al., 2020).

The current opinion about coordination of growth processes and divisions (Sablowski, 2016) stressed the role of individual cell characteristics and intercellular interactions in these processes. Optical microscopy is a valuable method for obtaining the structural characteristics on the subcellular resolution. For example, this approach allows studying the ultrastructural features of the cell wall (Yarbrough et al., 2009), which enables us to assess cellular biomechanics indirectly. The combination of large-scale annotated image datasets and deep learning approaches is a promising technique for annotating physical, morphological, and tissue grading cellular properties (Fricker, 2016; Biswas and Barma, 2020).

The cell wall’s mechanical parameters deserve special attention since they determine features of the growth process (Bidhendi and Geitmann, 2016), and therefore is incredibly important for modeling plant morphogenetic systems. In addition to assessing the thickness of the cell wall (Krzesłowska et al., 2019), modern approaches make it possible to evaluate its composition and mechanical parameters. For example, probe microscopy can assess the spatial composition of polysaccharide filaments on the surface of living tissues (Zhang et al., 2016), and Raman microscopy can produce data on the composition and ultrastructure of the cell wall on sections of organs in the usual (Zeise et al., 2018) and confocal modes (Gierlinger et al., 2012). The ultrastructure of cell walls as well as tissues and organs can be studied with a 3D electron microscope (Kremer et al., 2015). All these methods make it possible to assess biomechanical parameters within organs and serve as the basis to improve the simulation modeling of growth processes.

Therefore, the next important step is integrating the structure model with the cell parameters that mark the individual and group characteristics of cells (Figure 3). Many characteristics of the nucleus, organelles, and cell walls can be identified at the scale of an entire organ using approaches of protein immunolocalization, expression of reporter constructs that mark certain cellular features, as well as using methods to increase the resolution of microscopy (Figure 3).

The data on the frequency of mitoses along the root (Pasternak et al., 2017; Lavrekha et al., 2020) provides insight into the dynamics of replenishment of cell files and the size zones, where cell divisions occur. Also, cells in S-phase can be identified by incorporating labeled nucleotide analogs (Pasternak et al., 2017). The passage of the cell cycle phases is closely associated with the cell fate specification (Roeder et al., 2012). The state of chromatin in cells of various types can be identified using immunolocalization (She et al., 2018) and shed light on cell activity. Visualization of the cytoskeleton can be done both by immunolocalization, staining with phalloidin, and, *in vivo*, using reporter genetic constructs (Zhang et al., 2020). These cells’ characteristics can be related to changes in gene groups’ expression in cells and are suitable for improving the integration of the structural model with single-cell transcriptomic data.

The distribution of various proteins in plant organ cells can also be determined (Sauer and Friml, 2010) and used for integration into a model. Proteins can be transporters that determine the fluxes of substances that deserve special attention; for example, the auxin membrane transporter PIN1 has a significantly uneven distribution over root cells and a polar arrangement on the cell surface (Omelyanchuk et al., 2016). It has also been shown that RNA molecules capable of being transported from tissue to tissue play an essential role in the regulation of biological processes in a plant, and their visualization within an organ is also possible (Luo et al., 2018).

Also, plasmodesmata play a unique role in the processes of intercellular symplastic transport and signaling in plant tissues (for comprehensive review, see Heinlein and Epel, 2004). Plasmodesmata are intercellular channels characterized by various states from open to closed (Crawford and Zambryski, 2001). Plasmodesmata behavior underlies the isolation of groups of cells in the tissue, called symplastic domains (Pfluger and Zambryski, 2001; Lucas and Lee, 2004; Yadav et al., 2014). Stress factors affect the formation of plasmodesmata (Fitzgibbon et al., 2013). The transport of mRNA and metabolites through the plasmodesmata affects the concentration of substances and gene expression levels inside particular cells (Lucas and Lee, 2004). Many non-cell-autonomous transcription factors and small RNAs are known to move through plasmodesmata between cells and regulate their interaction during development (Kragler, 2013; Yadav et al., 2014; Sevilem et al., 2015).

Transmission electron microscopy is the classical method for studying the morphology of plasmodesmata. Combined with light-based microscopy, it allows one to study the structure and distribution of plasmodesmata between cells of specific cell types (Nicolas et al., 2017). Also, the topology of plasmodesmata of contacting cells at organ scale can be studied using confocal and super-resolution microscopy (Fitzgibbon et al., 2010, 2013). In this sense, microscopy allows us to assess the location and topology of plasmodesmata and, therefore, identify the potential of local transport of substances through these transport channels, symplastic domains, and to assess the order of cell division. Thus, the organization and localization of transport channels inside the plant tissues are connected with the intracellular characteristics.

On the other hand, intracellular sensing processes contribute to intercellular signaling. For instance, there are special sensory plastids in epidermal and vascular parenchyma cells, which can cause a global systemic stress response in a plant (Beltrán et al., 2018).

The redox state of organelles is also an additional factor associated with developmental processes, ROS signaling, and antioxidant systemic plant cells (Bobrovskikh et al., 2020). In particular, the CellROX fluorescent reagent visualizes the oxidative potential of cells in a tissue (Kováčik and Babula, 2017).

Besides, mass spectrometry imaging and live single–cell mass spectrometry practically corresponds to single-cell metabolomics and makes it possible, for example, to mark the concentrations of secondary metabolites on the whole adult organ (Yamamoto et al., 2019). Such approaches can be combined with SC analysis of the expression of these metabolites’ biosynthetic enzymes and transporters. As a result, they provide a basis for modeling the distributed regulation of these processes at the tissue level (Figure 3). The most important polynucleotides, such as RNA, can also be detected at the level of single molecules (Huang et al., 2020), which allows direct integration into the structural model of the organ.

Modern imaging techniques allow access to the structural and physiological characteristics of cells in a whole organ manner. It provides ample opportunities to create, enrich, and verify structural models of plant organs and tissues. An important aspect is that many assessments can be carried out over time. Comparison of temporal dynamics in zones with active morphogenetic events will make it possible to track changes in cellular topology, and thus, to trace the nature of division (symmetric and asymmetric) and growth (isotropic and anisotropic), as well as to detect several mechanical features of the developing tissue (for example, the relative stiffness of different cell zones).

Thus, a large arsenal of available microscopic and imaging techniques allows obtaining high-quality multilevel data integrated into plant morphogenesis models. For example, there is a computational morphodynamics approach that allows formalizing quantitative data from morphometry measurements into a set of rules (Formosa-Jordan et al., 2018):

1. To set ODE, which describes the growth rate of individual cells using data from regulatory networks.
2. To set various rules for the geometry of division (periclinal/tangential divisions with different angles) according to mechanical constraints of intercellular vertex interactions.
3. To use the first two steps to calculate effective growth and final rate equation.

## 4. Cell-Based Modeling Approaches Reproducing Plant Tissue Morphogenetic Processes

### 4.1. Existing Models and Modeling Approaches

This section will discuss existing mathematical models describing the tissue organization and/or properties of individual cell types. While considering plant growth and developmental processes, researchers often highlight a unique role for the hormone auxin. For instance, in plant roots, auxin triggers cascades of events during development and morphogenesis, while other hormones (cytokinins, brassinosteroids, abscisic acid, gibberellins, and others) interact with auxin (Saini et al., 2013). Auxin is also an important regulator in developing shoot apical meristems in combination with cytokinins, gibberellic acid, and some transcriptional factors: WUSCHEL, ARR7/ARR15, ARF5 (Durbak et al., 2012). Mironova et al. (2012) demonstrated the effectiveness of the reverse fountain and the reflected flow mechanisms of PIN-associated transport in the root apical meristem. Comparison of different complexity models showed that a model that only describes auxin transport processes is insufficient for the reproduction of realistic patterns of morphogenesis, but adding an additional layer-specific regulation or layer-driven growth could help solve this problem (De Vos et al., 2014).

Simultaneously, the mechanical characteristics of tissues, which are determined through a complex interplay of genetic and physiological systems, are an essential component for describing the processes of morphogenesis. The feedback effects of mechanical interactions and stresses, which affect the regulation of proliferation patterns, are highlighted in Nelson et al. (2005). The experimental evidence of the mechanical stress approach’s consistency for plant tissue development is shown in the work of Uyttewaal et al. (2012). The transition from the linear models of hormonal transport to hybrid multicellular and multiscale models has excellent potential for predicting the emergent properties of the system (Voß et al., 2014). The basis for mechanical models of cell growth is the representation of multicellular tissues in vertex-based graphs with the calculation of the interaction forces between these elements. The equations binding the growth of plant cells with the rate of water absorption and the cell wall’s growth were first published in Lockhart’s work for the case of constant turgor pressure (Lockhart, 1965). In order to model growth in a more general case, Lockhart’s equations were extended, taking into account the change in turgor pressure as a result of reversible elastic deformation and transpiration processes in the Ortega model (Ortega, 2010). Within the framework of this approach, a linear leaf growth model was proposed (Zubairova et al., 2016). In addition, Newton’s First Law and Hooke’s Law can be used to describe cell growth and expansion, as was done in the recent work by Retta et al. (2020).

Unfortunately, most available auxin-related models are focused only on the transport processes in the root tissue and poorly explain the overall processes of growth and development (Morales-Tapia and Cruz-Raḿırez, 2016). However, several models combine both a mechanical approach and auxin transport processes. For example, there is a dynamic model that describes molecular mechanisms in conjunction with physical tension fields and auxin dynamics (Barrio et al., 2013). This model reproduces emergent patterns of morphogenesis from proliferative to transition and elongation zones. The study combining experimental data on the organization of the extracellular matrix and numerical simulations demonstrated that auxin plays an essential role in altering cells’ mechanical properties; this process involves the ABP1 and KATANIN 1 proteins (Sassi et al., 2014). Also, the advanced cell-based mathematical model describes the relationship between the concentration of morphogens and the cellular mechanistic properties in the developing apical shoot meristems (Banwarth-Kuhn et al., 2019).

Thus, the models of plant tissue morphogenesis put at the forefront three biological facts: (i) the dependence on intercellular hormonal signaling, (ii) the importance of the intracellular state and individual cellular characteristics, (iii) the relevance of mechanical stresses in intercellular interactions. Therefore, scRNA-seq technologies, microscopy, imaging techniques, and a range of complementary approaches to measuring cell mechanical properties (Banwarth-Kuhn et al., 2019; Bidhendi and Geitmann, 2019) can provide a complete picture of morphogenetic processes at the cellular level.

### 4.2. Available Software and Tools for Cell-Based Modeling

In general, elaborating mathematical models of morphogenetic processes could base on specialized software, which we discuss in this section. Researchers may also develop and implement their frameworks and algorithms using mathematical packages and general-purpose programming languages (Python, Mathematica, MATLAB). Three formalisms are most often used to build cell-based models: vertex-based, center-based (also called spring-based), and Cellular Potts models. Vertex-based models are often used to simulate plant tissue and make it possible to conveniently describe the dynamics of cell movements in cell ensembles taking into account mechanical constraints (for example, during morphogenesis). This formalism is implemented in the Cellzilla (Shapiro et al., 2013), VirtualLeaf (Merks et al., 2011) packages. In center-based models, cells are represented as dots with mass, connected by mechanical elements (springs). Banwarth-Kuhn et al. (2019) give an example of this formalism’s application to the description of growth processes in the shoot apical meristem. Cellular Potts models are often used to describe the processes occurring in animal tissues and tumor formation processes; this formalism is implemented in CompuCell3D (Swat et al., 2012). It is also possible to use the Voronoi tessellation formalism for modeling morphogenetic processes; e.g., see Romero-Arias et al. (2017).

Below we discuss available software, while a summary is presented in Table 2; for more details, see Supplementary Table 1.

**Table 2:** The most popular tools for cell-based plant tissue morphogenesis modeling.

**Name, reference, link**	**Spatial scale**	**Formalism**	**Examples**
Virtual cell (Moraru et al., 2008)	2D/3D	Kinetics, diffusion, flow, membrane transport, electrophysiology	Gajdanowicz et al., 2011; Onal et al., 2020
OpenAlea (Pradal et al., 2008)	2D/3D	Functional-structural plant models	Muraro et al., 2014
CellModeller (Dupuy et al., 2008)	2D	Biphasic systems; viscous yielding of the cell walls	Dupuy et al., 2010; Rudge et al., 2012
VirtualLeaf (Merks et al., 2011)	2D	Vertex dynamics model	van Mourik et al., 2012; De Rybel et al., 2014; De Vos et al., 2014
CompuCell3D (Swat et al., 2012)	2D/3D	Cellular Potts model	Hester et al., 2011; Swat et al., 2015
CellZilla (Shapiro et al., 2013)	2D	Vertex dynamics model	Nikolaev et al., 2013; Shapiro et al., 2015
LBIBCell (Tanaka et al., 2015)	3D	Lattice Boltzmann method for solving fluid and signaling processes	Stopka et al., 2019

Virtual Cell (Cowan et al., 2012; vcell.org) is an environment for modeling, analysis, and simulation of cellular processes, and it includes tools for gene network and for the integration of biological images. This package consists of distinct functional modules: rule-based networks, ODE, PDE and kinematics, stochastic simulations, parameter estimation and has the ability to integrate it into hybrid models. Users can define the model structure and the system automatically builds the code and compiles it. A detailed overview of this tool is given in Moraru et al. (2008). Also, there is a VCell extension for compartmental and spatial rule-based modeling (Blinov et al., 2017). The implemented models using VCell can have a different scale, for example, the model of potassium transport in plant vascular tissues (Gajdanowicz et al., 2011), and model of the paracrine-juxtacrine loop for breast cancer cells and macrophages (Onal et al., 2020).

VirtualLeaf package (code.google.com/archive/p/virtualleaf/, Merks et al., 2011) using a vertex-based approach (Nagai and Honda, 2001); the algorithm includes vertex motions at each step that minimize the Hamiltonian energy by the Monte Carlo algorithm. For each cell, an unstressed area is specified, corresponding to the cell’s state when the turgor pressure is balanced with the external pressure. For each cell wall element, the unstressed length is specified, corresponding to the length of the cell wall segment in the absence of turgor pressure. The balance between turgor pressure and the cell wall’s resistance can be described in terms of the generalized potential energy (Hamiltonian) calculated as the sum of all cells and cell wall elements, which is then minimized by the algorithm. The growth models of root were implemented using this framework (De Vos et al., 2014).

Cellzilla uses a vertex dynamics model for describing morphodynamics processes and takes into account morphogenetic regulation (http://cellzilla.info/, Shapiro et al., 2013). The cellular structure is represented by a list of three elements: a list of vertex coordinates, a list of edges consisting of pairs of vertex numbers, and a list of cells consisting of lists of edge numbers belonging to a cell. The interaction between morphogens and the transport flows in each cell is described in terms of chemical kinetics using the arrow notation of the Cellerator package (Shapiro et al., 2003). This software automatically constructs and solves a system of differential equations describing the dynamics of morphogens’ concentration in all tissue cells. Methods for constructing models of plant cell growth in CellZilla are described by Shapiro et al. (2013). Using this system, Nikolaev et al. (2013) constructed a model for *A. thaliana* shoot apical meristem structure maintenance.

CellModeller (haselofflab.github.io/CellModeller/; Dupuy et al., 2010) is a software with modular structure for 2-dimensional simulations. It can reproduce the intracellular dynamics of metabolites, intercellular transport processes, as well as cell mechanics using physical laws. This software can be used for modeling plant morphogenetic processes. For example, a simple morphogenetic system for the Coleochaete alga has been developed (Dupuy et al., 2010).

LBIBCell (Tanaka et al., 2015, https://tanakas.bitbucket.io/lbibcell/) was developed specifically to simulate morphogenetic processes in tissues. This tool uses the immersed-boundary concept (which describes cells as viscous fluid with elastic walls), coupled with the Lattice Boltzmann method. The model of biased epithelial lung growth was implemented using this tool (Stopka et al., 2019).

OpenAlea (Pradal et al., 2008) is an integrative platform that combines various computational frameworks. This platform’s main goal is the integration and mutual enrichment of experience in different sections of plant process modeling. This system is based on Python language and has a visual programming interface. For example, the OpenAlea package VPlants (https://team.inria.fr/virtualplants/) allows building models of tissue morphogenesis. This package was used in modeling vascular development in *A. thaliana* (Muraro et al., 2014).

CompuCell3D (Swat et al., 2012) is a C++ software for 3D modeling, which includes both graphical user and command-line interfaces. This system uses classical mechanics for describing cellular behavior according to mechanical constraints. Multicellular systems are described using the Cellular Potts model. The input data include the grid’s size, number of cells, cellular interactions, energy functions, and activator concentrations. The protocol for using this program to study cellular morphogenesis parameters is presented in Palm and Merks (2015). Most of the models elaborated with this software describe the development of animal tissues (Hester et al., 2011) and the processes of tumorigenesis (Swat et al., 2015).

Thus, the available software and methods are pretty diverse, and the choice of a particular tool depends on the specifics of the task at hand. Among these tools, it is necessary to highlight Cellzilla and VirtualLeaf as the most specific for describing plant morphogenesis processes. On the other hand, the development of new frameworks and algorithms, which depend on researchers’ ability to program, is a promising approach since it significantly expands the functionality and removes several restrictions on applying one or another formalism implemented in existing software.

### 4.3. Our Framework and Model Flowchart

In this section, we propose a general framework for modeling plant morphogenetic processes based on various biological data. This kind of model should include two main data sources: scRNA-seq and tissue imaging data; besides, SC metabolomics and cell wall stiffness studies can serve as additional data sources. For plant organ growth modeling, the accurate description of processes on the cellular level is essential since this level combines molecular regulation with hormonal regulation, cell division, and reproduction processes (De Vos et al., 2012).

Mathematically, events occurring in plant tissues and cells can be classified into continuous and discrete ones. The first ones include the processes of metabolism, growth, transport and development of cells. Discrete events, on the other hand, include processes such as birth (or emergence), division, death, and change of cellular state. Individual cells’ metabolic characteristics are influenced by their genotype and developmental stage, which would be described by single-cell transcriptomics approaches. The nature of the proposed framework is hybrid since it combines different mathematical formalisms and modules: (i) ODE/PDE equations for describing the dynamics of substances and morphogens inside the cell and the processes of intercellular transport, (ii) discrete events occurring during the onset of threshold conditions (for example, cell division when a specific cell area is reached, or cell differentiation at a hormone concentration above the threshold), (iii) the biophysical laws of mechanical interactions between cells (such as Ortega’s approach Ortega, 2010 or Newton’s and Hooke’s laws Retta et al., 2020). In this sense, scRNA-seq data helps measure individual characteristics of cell populations (which characterize system dynamics), while microscopy should help to define geometrical patterns and “rules” (e.g., division geometry or dividing plane orientation). These steps will help to create hybrid models with tissue/cellular resolutions.

The usefulness of such a hybrid approach in describing ecological systems was described in the work of Vincenot et al. (2011). In particular, the combination of discrete and continuous phenomena is a natural property of multicellular systems, and such hybrid frameworks allow researchers to make more realistic simulations *in silico*. Van Liedekerke et al. (2015) described the advantages and disadvantages for different types of agent-based models of tissue mechanics and noted that hybrid models could reproduce spatial resolution, physical aspects of interactions, cell shapes diversity. Osborne et al. (2017) compared different approaches to cell-based modeling using typical cases of the described processes; the authors noted that the vertex-based approach, in contrast to others, allows one to simulate boundary conditions in proliferation processes effectively. This feature allows us to consider this method as the most promising for modeling the root apical meristems, which has more severe mechanical restrictions for growth than leaf and shoot tissues. For modeling leaf and shoot tissues, for example, it is possible to use the Voronoi tessellation or overlapping spheres modeling approach described in Osborne et al. (2017).

**Figure 4:**
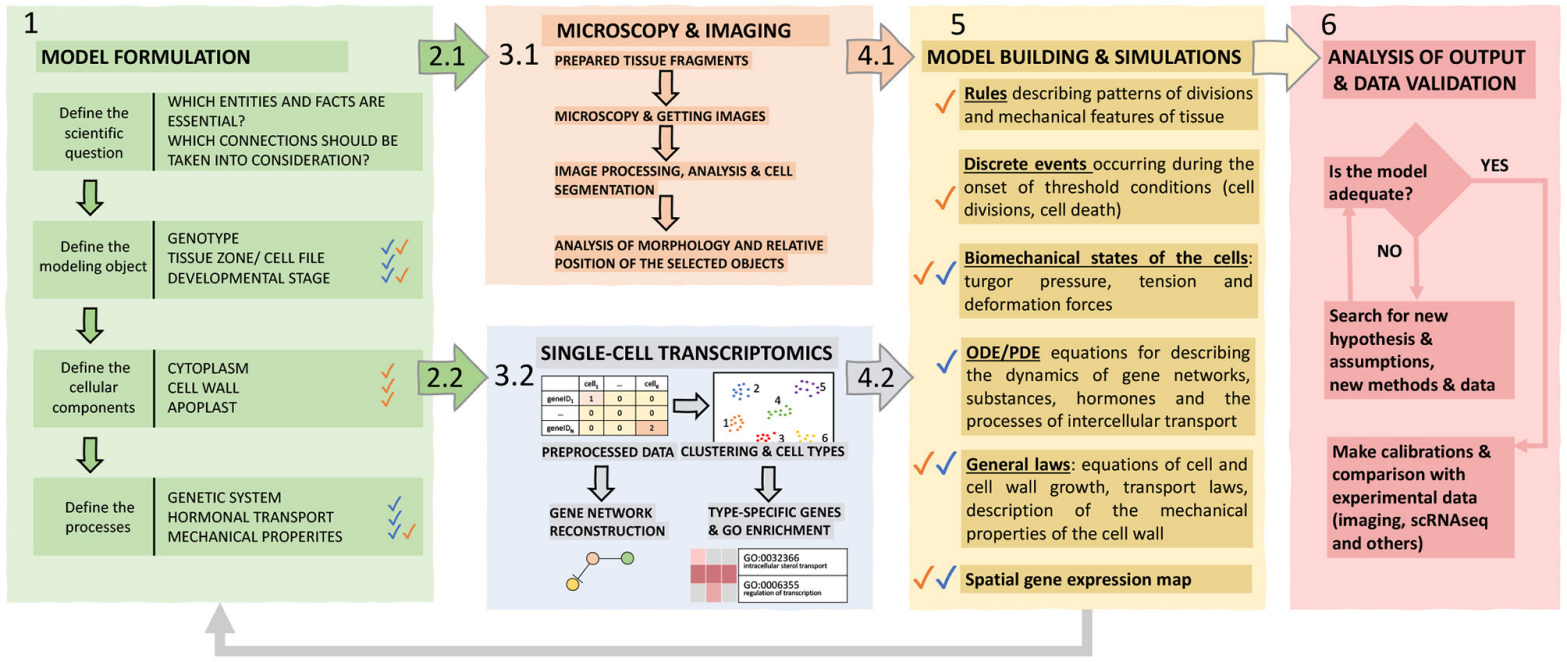
The proposed hybrid framework for cell-based models construction. The framework includes six functional blocks explained in the text. Individual blocks are marked with corresponding colors; colored arrows indicate the transition between blocks. Blue and orange checkmarks indicate information related to single-cell and imaging data, respectively.

Thereby, we assume the use of such a hybrid approach complementary to modern research due to its multilevel nature; it combines SC transcriptomic and microscopy data into a cell-based modeling framework. Below in the text and in Figure 4, we outline the main stages of our framework that must be taken into account.

1. The posed biological problem determines the structure of the model. A modeler should define a biological system’s properties, its elementary subsystems, and connections between these elements, which are significant to reproduce them in the model. Based on these decisions, it is necessary to determine the main properties of the simulated object: genotype, organ, tissue zone, stage of development. Since a cell is a crucial element for describing the processes of plant morphogenesis, the next step is to find out which cellular structures will be reproduced in the model to determine the formalism used to describe them and the equations for growth and the rules of division. Then, it is necessary to decide on the objects at the molecular level to be considered, in particular the genetic systems of interest, to find out whether it is required to consider transport processes for morphogens (for example, hormones), and also to decide whether it is necessary to take into account the biomechanics of cells for the modeled system.
2. Designing experiments to obtain imaging (2.1) and scRNA-seq (2.2) data based on the given aim. For imaging (2.1), it is essential to choose a suitable plant portion and microscopy technology and determine whether it is necessary to track the dynamics of development of a given fragment of tissue and for which interval of time. For scRNA-seq (2.2), it is important to make sure that the process of isolation of protoplasts and their analysis will not be limited due to the structure of the tissue and/or organ of the plant, imperfections, and shortcomings of the available methods, otherwise, this technique will have to be worked out and improved to an acceptable level.
3. Perform the experiments and produce data. (3.1) It is necessary to prepare (for example, fix and stain) a target tissue fragment, get images, process and analyze them (manually or using plugins), and digitize the resulting patterns to build a structural model of the tissue/organ and identify morphogenetic rules for incorporation into a computational model. (3.2) While obtaining and analyzing scRNA-seq data, special care should be taken to ensure that the research aim is as close as possible to the intended modeling goals. Care should be taken to avoid contamination with cells of those classes that are not needed and so that for most of the required cells, it would be possible to analyze the molecular systems required for the model. Besides, scRNA-seq-based approaches for the reconstruction of gene networks of the corresponding processes have high potential.
4. Analyze experimental data. Experimental results at cell and tissue level have to be analyzed in order to derive key parameters to be used in the model formulation in terms of cellular characteristics (4.1) and molecular processes (4.2) for all the considered cell types.
5. Systematic assembly of the hypotheses, available data and mathematical formalization into a single hybrid model, which consists of the following blocks: (1) ODE / PDE equations for describing the dynamics of substances and morphogens inside the cell and the processes of intercellular transport, (2) discrete events occurring at the onset of threshold conditions (for example, cell division when a specific cell area is reached, or cell differentiation at a hormone concentration above the threshold), (3) biomechanics interactions between cells (4) agent-based rules describing patterns of divisions and mechanical features of the tissue.
6. Validation and verification of models is based on their success in reproducing the behavior of real biological phenomena that can be evaluated experimentally. In this sense, it can be useful to return to the stage of morphometry and compare the dynamics of tissue development with simulations and study in detail the molecular organization of the subsystems described in the model.

In general, the proposed approach is universal for describing any morphogenetic system; however, the pipeline described above may differ in some steps for each specific case, while some of them could be eliminated. In particular, plant tissue morphodynamics is context-dependent due to mechanical interactions inside cell ensembles and the transport of morphogens through plasmodesmata, which is confirmed by numerous studies (Crawford and Zambryski, 2001; Heinlein and Epel, 2004; Lucas and Lee, 2004; Kragler, 2013; Yadav et al., 2014; Sevilem et al., 2015; Luo et al., 2018). At the same time, models for morphodynamics of animal tissues with strong neighborhood structures could include analogous mechanisms modified to consider cell adhesion processes. For example, this approach is applicable to model the processes of animal epithelial or tumor growth (Interian et al., 2017).

## 5. Conclusions and Future Challenges

Post-genomic technologies made it possible to obtain detailed information about processes at genomic and transcriptomic levels using SC and whole tissue RNA sequencing technologies. Besides, the existing abundance of microscopy methods allows high-quality characterization of morphology and physiology at the level of extended fragments of tissues and organs. However, microscopy approaches do not allow to perform quantitative assessments of important intracellular characteristics, such as concentrations of substances and metabolites. SC metabolomics approaches for plants, which are beyond this review’s scope, still remain overshadowed, although significant developments have been made in mass spectrometry approaches for such kind of analyses (de Souza et al., 2020). Gilmore et al. (2019) discuss the latest advances in mass spectrometry imaging: matrix laser desorption ionization (MALDI) and secondary ion mass spectrometry (SIMS), which have a high potential for assessment of metabolism at subcellular spatial resolution. The development of these methods will allow metabolomics to achieve the same spatial resolution level as SC transcriptomic. The review of Bidhendi and Geitmann (2019) presents the main features and possibilities of measuring the cell wall’s mechanical properties: indentation technique, tensile test, acoustic microscopy, fracture measurements, and microfluidics. The authors emphasize that multiscale *in silico* mechanical modeling has excellent potential for the field and could help obtain a unified understanding of mechanical behavior across different scales.

To date, the methods and technologies necessary to obtain various experimental data for plant morphogenesis models have reached a balance and are mostly consistent with each other in terms of power, productivity, and spatial resolution. The community of mathematical biologists and programmers faces crucial theoretical challenges and is creating efficient computational frameworks capable of large-scale numerical simulations involving cellular ensembles of several thousands of cells. Such models will provide more accurate resolution and realism in the description of morphogenetic processes. Examples of optimization works are the algorithm of Jeannin-Girardon et al. (2015), and graphics processing units (GPU) accelerated framework for 3D cellular growth and division models (Madhikar et al., 2018). Moreover, declarative modeling perspectives concerning morphogenetic processes are considered (Mjolsness, 2019), which potentially will help formalize mathematical calculations at higher levels compared to general-purpose programming languages.

The widespread development of SC technologies in the future could serve as a driver for other areas of cellular and developmental biology of plants (Libault et al., 2017). However, we have an urgent need for data integration to successfully apply the technology, in particular at tissue level with its organization’s peculiarities as an emerging system. Besides, an increased availability of SC data can stimulate the development of methods and modeling concepts at cellular and tissue levels, which will open the way for the binding of multi-omics characteristics for individual cell types and the observed phenotype.

On the other hand, it is necessary to verify the emerging issues related to the interpretation and analysis of SC data using advanced microscopy and *in silico* biology. In this sense, one of the most urgent problems of SC sequencing is the reverse reconstruction of the spatial position of cells based on corresponding transcriptome expression. Searching for major regulatory genes that characterize certain cell lines will be a critical step to solve this problem. Also, cell-based models of morphogenesis could help interpret and integrate SC and imaging data, making the reasoning more transparent and establishing an understanding of essential parameters and mechanisms for the described systems.

Summarizing all of the above, we have found the following key features related to SC-technologies that need to be addressed:

1. Some limitations are still present in the phases of integration, analysis, and interpretation of data.
2. Only a limited set of plant species and organs is suitable for obtaining transcriptome and structural data with cellular resolution.
3. There is a need for a more precise reconstruction of scRNA plant atlases.

The task of elaborating and analyzing *in silico* models of morphogenesis, due to the complexity of the studied systems and computational limitations, are non-trivial. Thus, cell-based models, which use a hybrid formalism, could effectively combine our knowledge on different levels and help tackle the complexity of the system. However, the current problem of the large number of dimensions of the initial SC data should be solved by applying preprocessing and filtering algorithms, as well as for the reconstruction of related gene networks. Thereby, model formulation and numerical experiments *in silico* could be applied using only the essential part of the initial high-dimensional SC data. Such reduction should aim to contain data on gene expression changes and metabolites concentrations, which determine the different cellular states.

## Author Contributions

AB prepared the draft text of the manuscript and figures. UZ and AD developed the concept and edited the manuscript and figures. AB, AD, and UZ finalized the specific parts. FG developed a scheme for single-cell tools describing. FG, FC, and SM participated in discussing the concept of the article, advised writing sections of the manuscript and design of figures. All authors contributed to the manuscript and approved the submitted version.

## Conflict of Interest

The authors declare that the research was conducted in the absence of any commercial or financial relationships that could be construed as a potential conflict of interest.

## Abbreviations

*A. thaliana*: *Arabidopsis thaliana* L.
SC: single-cell
SCS: single-cell sequencing
scRNA-seq: single-cell RNA sequencing
RNA-seq: RNA sequencing
t-SNE: t-distributed Stochastic Neighbor Embedding
UMAP: Uniform Manifold Approximation and Projection
LSM: Laser Scanning Microscopy
LS: Light-Sheet Microscopy
SPM: Scanning Probe Microscopy
SIM: Structured Illumination Microscopy
3D-SEM: 3-Dimensional Scanning Electron Microscopy
ODE: Ordinary Differential Equation
PDE: Partial Differential Equations.
